# First Discovery of Acetone Extract from Cottonseed Oil Sludge as a Novel Antiviral Agent against Plant Viruses

**DOI:** 10.1371/journal.pone.0117496

**Published:** 2015-02-23

**Authors:** Lei Zhao, Chaohong Feng, Caiting Hou, Lingyun Hu, Qiaochun Wang, Yunfeng Wu

**Affiliations:** 1 College of Plant Protection, State Key Laboratory of Crop Stress Biology for Arid Areas, Key Laboratory of Crop Pest Integrated Pest Management on Crop in Northwestern Loess Plateau, Ministry of Agriculture, Key Laboratory of Plant Protection Resources and Pest Management, Ministry of Education, Northwest A&F University, Yangling, Shaanxi, China; 2 College of Horticulture, State Key Laboratory of Crop Stress Biology for Arid Areas, Key Laboratory of Crop Pest Integrated Pest Management on Crop in Northwestern Loess Plateau, Ministry of Agriculture, Key Laboratory of Plant Protection Resources and Pest Management, Ministry of Education, Northwest A&F University, Yangling, Shaanxi, China; National Institute of Infectious Diseases, JAPAN

## Abstract

A novel acetone extract from cottonseed oil sludge was firstly discovered against plant viruses including *Tobacco mosaic virus* (TMV), *Rice stripe virus* (RSV) and *Southern rice black streaked dwarf virus* (SRBSDV). Gossypol and β-sitosterol separated from the acetone extract were tested for their effects on anti-TMV and analysed by nuclear magnetic resonance (NMR) assay. In *vivo* and field trials in different geographic distributions and different host varieties declared that this extract mixture was more efficient than the commercial agent Ningnanmycin with a broad spectrum of anti-plant-viruses activity. No phytotoxic activity was observed in the treated plants and environmental toxicology showed that this new acetone extract was environmentally friendly, indicating that this acetone extract has potential application in the control of plant virus in the future.

## Introduction

Viral diseases have long threatened sustainable development of agriculture [1, 2]. Over the last decades, efforts have been exerted to develop strategies for efficient control of them [3–5]. Biological and chemical methods have great potential for this purpose. Ningnanmycin is one of the most successfully registered anti-plant viral agent and displayed 56.0% *in vivo* curative effect at 500 μg mL^-1^ [3]. Ribavirin, another widely used plant viral inhibitor, has lower than 50% inhibitory effects at 500 μg mL^-1^ [3]. Lower cure rates (30–60%) and probably negative effects on environments of these antiviral agents limit their wider applications. Many efforts still have to be done to develop highly efficient and environmentally friendly antiviral agents.

Nowadays, natural-product-based antiviral agents are more popular than those produced by chemosynthesis. Since plants have evolved multiple mechanisms to resist pathogens by producing secondary metabolites with antimicrobial activities, natural products from plants have been proved to be a rich resource of antiviral agent. Moreover, nature-derived plant substances have less negative impact on the environment, as they are readily broken down and have short-lasting residual periods [6, 7]. With the increasing worldwide concern on virus diseases, exploitation of new nature-derived anti-viral agents received increasing attentions.

*Tobacco mosaic virus* (TMV), the first plant virus to be discovered, has been listed among the top 10 plant viruses in molecular plant pathology [2], and maintained as a model virus for more than 110 years [8]. *Rice stripe virus* (RSV) and *Southern rice black streaked dwarf virus* (SRBSDV) are two of the most damaging viruses in rice [9, 10]. In our preliminary study, we found that acetone extract from cottonseed oil sludge displayed moderate antiviral activity against plant viruses. The aim of this study was, therefore, to analyze the antiviral activity of the acetone extract compare its effects with Ningnanmycin, effective compounds were separated from the acetone extract. Phytotoxic activity and environmental toxicology of the acetone extract were also tested.

## Materials and Methods

### Materials

In order to improve the concentration of bioactive compounds in the acetone extract, cottonseed oil sludge was used as the raw material, instead of cottonseed. It was purchased from Jingyang Sanqu oil factory in shaanxi province. TMV isolates were provided by the State Key Laboratory of Crop Stress Biology for Arid Areas, Northwest A&F University in the form of virus infected plants of *Nicotiana tabacum* L. cv. K326. *Nicotiana glutinosa* was used as a local lesion host and cultivated in the insect-free greenhouse at 23–25°C. The experiments could be conducted when the plants grow to 5–6 leaves (about 8 weeks old). For the field trials, Rice (*Oryza sativa* L.) infected with RSV and SRBSDV all the year around was used to test the antiviral activities of the acetone extract. The experiment was carried out in most of the main rice producing regions in China (Table 1).

**Table 1:** Antiviral activity of the acetone extract against *Rice stripe virus* (RSV) under field conditions.

Testing units and test site	Specific location	Contact Person	Host variety	Plot area for seeding	Compound	Dosage g ha^-1^	Average disease index	Average control effect (%)	Significant difference analysis 5%
Zhangzhou Academy of Agricultural Sciences, Zhangzhou, Fujian	Zhangzhou City Longwen Area Chaoyang Town	Shunzhang Wu	Teyou Duoxi 1	20 m^2^	Acetone extract	300	2.46	51.01	c
						450	1.66	63.83	b
						600	1.24	75.06	a
					8% Ningnanmycin	750	1.69	66.32	b
					Control		4.89		-
Institute of Plant Protection, Fujian Academy of Agricultural Sciences Longyan, Fujian	Longyan City Shanghang County Huyang Town Huyang Village	Furu Chen	Shanyou 63	30 m^2^	Acetone extract	300	3.21	51.8	bA
						450	2.89	56.6	abA
						600	2.59	61.11	aA
					8% Ningnanmycin	750	2.4	63.96	aA
					Control		6.66	-	-
Institute of Plant Protection, Jiangsu Academy of Agricultural Sciences, Guanyun Jiangsu	Lianyungang City Guanyun County	Zhonghou Chen	Xindao 18	25 m^2^	Acetone extract	300	1.13	50.52	b
						450	1.14	56.57	a
						600	0.94	61.3	a
					8% Ningnanmycin	750	1.08	55.45	ab
					Control		2.77	-	-
College of Agronomy, Jiangxi Agricultural University, Jiangxi	Jiujiang City Pengze County Qianshan Town Minjiaqiao Village	Jianmei Xiong	Liangyoupeijiu	60 m^2^	Acetone extract	300	2.4	65.07	a
						450	2.18	68.12	a
						600	2.01	70.79	a
					8% Ningnanmycin	750	2.35	66.18	a
					Control		7.1	-	-
College of Agronomy, Jiangxi Agricultural University, Jiangxi	Jiujiang City Pengze County Qianshan Town Minjiaqiao Village	Jianmei Xiong	Liangyou 6326	60 m^2^	Acetone extract	300	2.45	67.19	a
						450	2.32	68.74	a
						600	2.09	72.19	a
					8% Ningnanmycin	750	2.38	67.8	a
					Control		7.54	-	-
Hunan plant Protection Institute, Hunan	Changsha City Changsha County Langli Town	Yong Liu	Jinyou 207	30 m^2^	Acetone extract	300	2.64	64.42	b
						450	2.09	71.73	ab
						600	1.48	80.23	aA
					8% Ningnanmycin	750	2.01	72.79	ab
					Control		7.46	-	-
Hunan plant Protection Institute, Hunan	Changsha City Changsha County Langli Town	Yong Liu	Jinyou 207	30 m^2^	Acetone extract	300	3.38	63.66	cB
						450	2.78	70.42	b
						600	2.02	78.31	aA
					8% Ningnanmycin	750	2.71	71.07	b
					Control		9.49	-	-

### Extract of the antiviral agent

The acetone extract was drawn by acetone from cottonseed oil sludge, which was the precipitate of cottonseed oil by adding sodium hydrate (15 baume degree). One kilogram of cottonseed oil sludge was dissolved in three liter of acetone. The mixture was agitated and kept at room temperature for 3 hour, and then filtrated. The residual filter was extracted again in the same way as described above and finally the two extracts were mixed together.

### Separation of active compound

The 10 litre acetone extract from cottonseed oil sludge was evaporated and dissolved in water, and extracted with the same volume of petroleum ether, ethyl acetate and n-butanol, respectively. The ethyl acetate extracts and n-butanol extracts were isolated by means of silica gel (Qingdao Haiyang Chemical Co., Qingdao, China) and reverse-phase (RP-18) column chromatography (YMC-Triart C18) (YMC America, Inc. Pennsylvania, America), and were further purified by Sephedex LH-20 (40–70 μm) (Amersham Pharmacia Biotech AB, Uppsala, Sweden). Thin-layer chromatography was conducted on silica gel plates GF254 (Qingdao Haiyang Chemical Co., Qingdao, China). The products were unequivocally characterized by NMR AVANCE III (Switzerland), using Bruker Topspin software version 3.0 a.

### Assays of antiviral activity in the greenhouse

By using the half-leaf method, we tested inhibition activities of the extract mixture and also the individual compounds gossypol and β-sitosterol, which were purchased from Sigma. Single or combination of gossypol and β-sitosterol were all dissolved with acetone and diluted with water at the concentrations of 100 and 500 μg mL^-1^. Ningnanmycin (8%) was diluted with water at the concentration of 500 μg mL^-1^ according to the manufacturer’s instructions. The extract mixture was dissolved with acetone and diluted with water at the concentrations of 100 and 500 μg mL^-1^.

TMV was purificated by using Gooding’s method [11] and *Nicotiana tabacum* L. cv. K326 inoculated with TMV was used. The upper leaves were selected, ground in phosphate buffer, and then filtered through a double-layer pledget. The filtrate was centrifuged at 10,000 g, treated twice with PEG, and centrifuged again. The whole experiment was carried out at 4°C. Absorbance values were estimated at 260 nm by using an ultraviolet spectrophotometer. TMV diluted to 6 ×10^–3^ mg mL^-1^ was used for the following experiment. Virus concentration was defined as the following formula.

$$Virus\;concentration\;=\;\frac{\left(A_{260}\times dilution\;ratio\right)}{E_{1\;cm}^{0.1\%,\;260\;nm}}$$

Inactivation effect, protective effect and curative effect were tested as follows. Inactivation effect of acetone extract, β-sitosterol, gossypol and Ningnanmycin against TMV in vivo. The purified TMV (6 ×10^–3^ mg mL^-1^) was mixed with acetone extract, β-sitosterol, gossypol or Ningnanmycin solution at the same volume or concentration, respectively. After 30 min the mixture was rubbed on the left side of the leaves of tobacco (*Nicotiana glutinosa*), whereas the right side of the leaves was inoculated with the mixture of the solvent acetone and the purified TMV as control. The local lesion numbers were recorded 4–5 days after inoculation. Each experiment was repeated three times.

Protective effect of the acetone extract, β-sitosterol, gossypol and Ningnanmycin against TMV in vivo. The solution of the acetone extract, β-sitosterol, gossypol or Ningnanmycin was smeared on the left side respectively and the solvent acetone serving as control on the right side of *Nicotiana glutinosa* leaves of the same ages. After 12 h the leaves were then inoculated with the purified virus (TMV at 6 ×10^–3^ mg mL^-1^), which was previously scattered with silicon carbide. The leaves were then washed with water. The local lesion numbers were recorded 4–5 days after inoculation. Each experiment was repeated three times.

Curative effect of the acetone extract, β-sitosterol, gossypol and Ningnanmycin against TMV in vivo. Purified TMV at 6 ×10^–3^ mg mL^-1^ was inoculated on the whole leaves of tobacco (*Nicotiana glutinosa*) of the same age. Then the leaves were washed with water and dried. The tested solutions of the acetone extract, β-sitosterol, gossypol or Ningnanmycin were smeared on the left side, and the solvent acetone was rubbed on the right side as control. The local lesion numbers were then counted and recorded 4–5 days after inoculation. Each experiment was repeated three times.

The in vivo inhibition rates of the compounds were then calculated according to the following formula. Inhibition rate (%) = [(C-T)/C] × 100%, where C is average local lesion No. of control, and T is average local lesion No. of drug-treated tobacco leaves.

Analysis of inhibition effect of the acetone extract on TMV by Dot-ELISA. Leaves of *N*. *tabacum* cv. K326 were prepared according to the Leaf Disk Method. Growing leaves of 8-week-old tobacco were mechanically inoculated with equal volumes of purified TMV (30 μg mL^-1^). After 72 h, leaves that were smooth and thin and without major veins were cut off and floated in the different concentrations of the acetone extract (100 μg mL^-1^, 200 μg mL^-1^, 400 μg mL^-1^ and 800 μg mL^-1^), Ningnanmycin (200 μg mL^-1^) or the solvent acetone (positive control), respectively, and were kept in a culture chamber at 25°C for 48 h. Then leaves were collected and the Dot-ELISA assay was used to analyze the coat protein of TMV by using a commercial kit according to the manufacturer’s instructions (Neogen, Beijing, China).

### Antiviral activity of the acetone extract in the field trials

The experiment was carried out by different institutes or universities in several outdoor experimental plots in the south of China (Tables 1 and 2), including Experimental Fields of Institute Zhangzhou Academy of Agricultural Sciences; Institute of Plant Protection, Fujian Academy of Agricultural Sciences; Institute of Plant Protection, Jiangsu Academy of Agricultural Sciences; College of Agronomy, Jiangxi Agricultural University; Hunan plant Protection Institute; Pesticides Administration of Jiangxi; College of Biosafety Science and Technology, Hunan Agricultural University. All the tested fields were private lands which belong to different institutes or universities and used especially for field test, specific locations and Contact person were shown in Tables 1 and 2. No specific permissions were required and no endangered or protected species were involved throughout the tested fields. In these fields, rice was naturally co-infected with *Rice stripe virus* (RSV) and *Southern rice black streaked dwarf virus* (SRBSDV) throughout the year. Twenty days after transplanting, the compounds (the extract mixture and Ningnanmycin) were sprayed twice with an interval of 14 days. Three dosages were used for the acetone mixture at 300, 450 and 600 g ha^-1^, compared with 8% Ningnanmycin at 750 g ha^-1^. Both of these compounds were diluted with 750 L water per hectare. Water alone was used as negative control. Each experiment was repeated four times and the area of each district was varied from 20–60 m^2^, depending on different geographical locations in the south of China (Tables 1 and 2). The survey was carried out following the national standard GB/T 17980. 19–2000 called ‘pesticide, guidelines for the field efficacy trials, fungicides against leaf diseases of rice’. In each plot, 50 plants for each of 5 points were selected in a diagonal line. Flag leaf, top second leaf and top third leaf were investigated in each plant and recorded as total leaf number, number of diseased leaves and disease grade. The classification of disease conditions were described as follows. Grade 0: no disease found in the whole plant; Grade 1: percentage of lesion size out of the total leaf area is lower than 1% and the length of lesion length is shorter than 0.3 cm; Grade 3: percentage of lesion size out of the total leaf area is between 2–5% and the lesion length is longer than 0.3 cm; Grade 5: percentage of lesion size out of the total leaf area is between 6–25%. Some lesions connect and are longer than 1 cm; Grade 7: percentage of lesion size out of the total leaf area is between 26–50%. Most of the lesions connect and are longer than 1 cm; Grade 9: percentage of lesion size out of the total leaf area is more than 50%. Lesions connect and large area of the leaf necroses. The disease index and control effects were calculated according to the following formulas.

Disease index=[∑(leaf number of every level of diseased plants×value of relative series)/(total leaf number of investigated plants×9)]×100

Control effect(%)=[(disease index of control plants-disease index of agent treated plants)/disease index of control plants]×100

DMRT method was applied to do variance analysis for determining the significance difference between every treatment.

**Table 2:** Antiviral activity of the acetone extract against *Southern rice black streaked dwarf virus* (SRBSDV) under field conditions.

Testing units and test site	Specific location	Contact Person	Host variety	Plot area for seeding	Compound	Dosage g ha^-1^	Average disease index	Average control effect	Significant difference analysis 5%
Zhangzhou Academy of Agricultural Sciences, Zhangzhou, Fujian	Zhangzhou City Longwen Area Chaoyang Town	Shunzhang Wu	Teyou Duoxi 1	20 m^2^	Acetone extract	300	2.11	43.67	c
						450	1.59	57.33	b
						600	1.18	67.85	a
					8% Ningnanmycin	750	1.31	63.90	ab
					Control		3.71	-	-
Institute of Plant Protection, Fujian Academy of Agricultural Sciences Longyan, Fujian	Longyan City Shanghang County Huyang Town Huyang Village	Furu Chen	Teyou 175	30 m^2^	Acetone extract	300	5.5	55.09	b
						450	4.0	67.34	ab
						600	3.25	73.46	a
					8% Ningnanmycin	750	3.75	69.38	a
					Control		12.25	-	-
Institute of Plant Protection, Jiangsu Academy of Agricultural Sciences, Guanyun Jiangsu	Lianyungang City Guanyun County	Zhonghou Chen	Xudao 3	25 m^2^	Acetone extract	300	0.8	48.61	c
						450	0.72	55.64	b
						600	0.68	62.16	a
					8% Ningnanmycin	750	0.7	56.16	b
					Control		1.85	-	-
College of Agronomy, Jiangxi Agricultural University, Jiangxi	Ji’an City Wan’an County Luotang Town Fengjia Village	Jianmei Xiong	Liangyou 6326	60 m^2^	Acetone extract	300	2.86	67.68	b
						450	2.57	70.97	ab
						600	2.36	73.43	a
					8% Ningnanmycin	750	2.72	69.49	ab
					Control		8.91	-	-
Pesticides Administration of Jiangxi, Jiangxi	Ji’an City Wan’an County Furong Town Guangming Village	Minghui Xiao	Rongyou 308	30 m^2^	Acetone extract	300	6.0	65.6	c
						450	4.6	75.2	b
						600	3.5	80.8	a
					8% Ningnanmycin	750	4.5	74.4	b
					Control		21.3	-	-
Hunan plant Protection Institute, Hunan	Changsha City Changsha County Langli Town	Yong Liu	Jinyou 207	30 m^2^	Acetone extract	300	3.5	69.09	cB
						450	2.75	78.82	ab
						600	2.25	83.99	aA
					8% Ningnanmycin	750	3.25	74.85	bc
					Control		11.5	-	-
College of Biosafety Science and Technology, Hunan Agricultural University, Hunan	Zhuzhou City Yanling County Luyuan Town Xitang Village	Tuyong Yi	Tianyou 998	30 m^2^	Acetone extract	300	3.8	57.0	b
						450	3.0	65.9	a
						600	2.8	68.5	a
					8% Ningnanmycin	750	3.1	64.3	a
					Control		8.8	-	-

### Phytotoxic activity and environmental toxicology

For the study of phytotoxic activity, the acetone extract was diluted to 500 μg mL^-1^, sprayed on the leaves of the tested tobacco and rice, and regularly observed. Environmental toxicology was carried out by Institute of Plant Protection (IPP), Chinese Academy of Agricultural Sciences (CAAS) from 2012 to 2013.

## Results

### NMR analysis

Finally, two bioactive compounds were obtained from ethyl acetate extract. Both the compounds were identified by physical, chemical properties and organic spectroscopy (^1^H NMR, ^13^C NMR), as well as contrast with the standard compounds. Their structures were identified as Gossypol and β-sitosterol. NMR data were shown in Tables 3–5.

**Table 3:** ^13^C-NMR and ^1^H-NMR data of gossypol.

Position C	δ_C_	Position H	δ_H_	DEPT	Couplings in HMBC
C-1,C-1’	151.32	1-OH, 1’-OH	5.821, 5.862(s)	C	
C-2,C-2’	114.83			C	C_2_, H_4_C_2_, 1-OH
C-3,C-3’	115.78			C	C_3_, H_4_ C_3_, H_14_
C-4,C-4’	118.52	H_4_, H_**4**_’	7.901, 7.679(s)	CH	C_4_, H_14_
C-5,C-5’	134.41			C	C_5_, H_4_C_5_,6-OH, C_5_, H_11_C_5_, H_12_
C-6,C-6’	143.5	6-OH, 6’-OH	6.467, 6.312(s)	C	C_6_, 6-OH, C_6_H_11,_ C_6_, 7-OH
C-7,C-7’	156.91	7-OH, 7’-OH’	15.522, 14.753(s)	C	C_7_, 7-OH, C_7_, 6-OH, C_7_, H_15_
C-8,C-8’	111.28			C	C_8_, H_15_C_8_, 7-OH
C-9,C-9’	151.04			C	C_9_, H_4_C_9_, 1-OH
C-10,C-10’	130.08			C	C_10_, H_4_C_10_, H_11_C_10_, H_13_
C-11,C-11’	27.89	H_11_, H_11_’	3.913, 3.815(m)	CH	C_11_, H_12_
C-12,C-13	20.26	H_12_, H_13_	1.567, 1.543(d)	CH_3_	C_12_, H_11_
C-12’,C-13’	19.92	H_12_’, H_13_’	1.661,1.632(d)	CH_3_	C_13_, H_11_
C-14,C-14’	20.14	H_14_, H_14_’	2.126, 1.139(s)	CH_3_	C_14_, H_4_
C-15,C-15’	200.24	H_15_, H_15_’	11.237, 10.768(s)	CH	C_15_, 7-OH

**Table 4:** ^1^H-NMR data of β-sitosterol.

Position H	δ_H_
H-3	3.50 (m)
H-6	5.348 (d)
H-14	2.279 (s)
H-18,18’,18”	1.021 (s)
H-19,19’,19”	0.677 (s)
H-21,21’,21”	0.916 (m)
H-26,26’,26”	0.842 (m)
H-27,27’,27”	0.861 (m)
H-29,29’,29”	0.771 (m)

**Table 5:** ^13^C-NMR data of β-sitosterol.

Position C	δ_C_	Position C	δ_C_
C-1	36.91	C-16	29.25
C-2	30.26	C-17	56.14
C-3	71.67	C-18	11.96
C-4	43.19	C-19	19.76
C-5	104.68	C-20	35.91
C-6	121.71	C-21	19.40
C-7	31.82	C-22	34.03
C-8	31.89	C-23	27.32
C-9	50.21	C-24	45.35
C-10	36.43	C-25	29.06
C-11	22.12	C-26	18.98
C-12	39.83	C-27	20.12
C-13	42.32	C-28	23.81
C-14	56.89	C-29	12.18
C-15	25.17		

### Anti-TMV activity in the greenhouse

**Fig 1:**
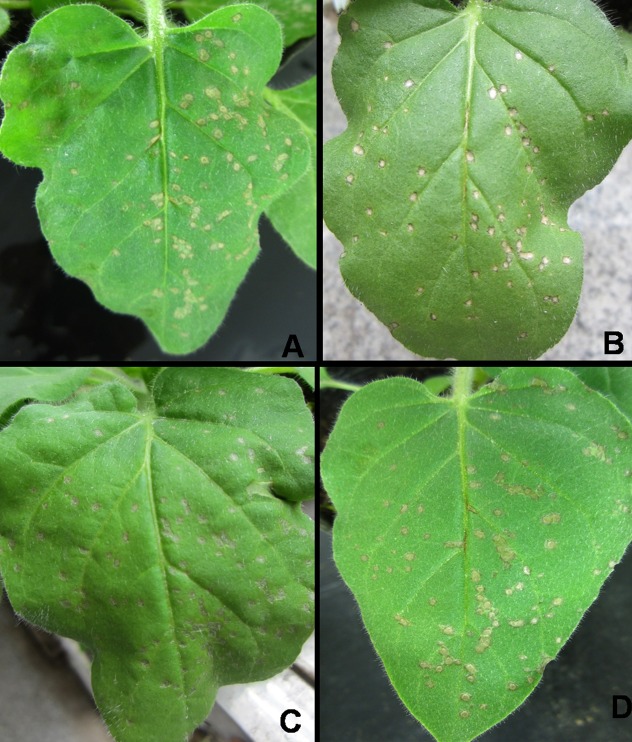
Comparison of curative effects of the acetone extract, β-sitosterol, gossypol and Ningnanmycin against TMV in *Nicotiana glutinosa* leaves. A: acetone extract at 500 μg mL^-1^; B: β-sitosterol at 500 μg mL^-1^; C: gossypol at 500 μg mL^-1^; D: Ningnanmycin at 500 μg mL^-1^. All the left side of the leaves were treated with the tested solutions and the right side with the solvent acetone as control.

As shown in Table 6 and Fig. 1, the acetone extract showed the highest anti-TMV activity during all the compounds tested, regardless of its concentration (100 or 500 μg mL^-1^). Antiviral activity of the acetone extract was much better than 8% Ningnanmycin, with inactivation effect at 71.5%, protection effect at 67.6% and curative effect at 71.2% for the extract mixture (Table 6), but with inactivation effect at 63.5%, protection effect at 61.7% and curative effect at 58.1% for Ningnanmycin (Table 6). When compared with its effective components gossypol and β-sitosterol, the acetone mixture also showed much better antiviral effect, even though higher concentrations (500 μg mL^-1^) of gossypol and β-sitosterol were used. Antiviral effect of gossypol was higher (inactivation effect at 57.5%, protection effect at 62.9% and curative effect at 54.4%) than that of β-sitosterol (inactivation effect at 40.2%, protection effect at 52.5% and curative effect at 45.6%) at the concentration of 500 μg mL^-1^.

**Table 6:** Anti-TMV activity of acetone extract, β-sitosterol, β-sitosterol gossypol mixture， gossypol, and Ningnanmycin.

Compounds	concentration (μg mL^-1^)	Inactivation effect (%)	Protection effect (%)	Curative effect (%)
acetone extract	100	64.1	61.4	56.3
	500	71.5	67.6	71.2
β-sitosterol	100	35.4	43.5	37.8
	500	40.2	52.5	45.6
^a^β-sitosterol and gossypol	100	53.3	55.6	49.7
	500	68.2	72.5	67.1
gossypol	100	47.2	41.6	43.5
	500	57.5	62.9	54.4
Ningnanmycin	500	63.5	61.7	58.1

^a^ Both of β-sitosterol and gossypol were at the concentration of 100 and 500 μg mL^-1^.

**Fig 2:**
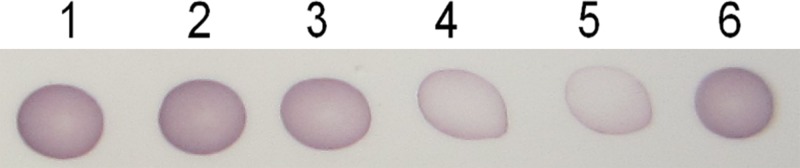
Inhibitory effect of the acetone extract and Ningnanmycin on TMV in tobacco tested by Dot-ELISA. 1: positive control, 2: acetone extract at 100 μg mL^-1^, 3: acetone extract at 200 μg mL^-1^, 4: acetone extract at 400 μg mL^-1^, 5: acetone extract at 800 μg mL^-1^, 6: Ningnanmycin at 200 μg mL^-1^.

Dot-ELISA assay (Fig. 2) shown that, signal of TMV in tobacco leaves gradually became weaker with the increased concentration of acetone extract from 100 μg mL^-1^ to 800 μg mL^-1^, indicating that the acetone extract may inhibit replication of TMV in plants.

### Antiviral activity in the field trials

Antiviral activity of the acetone mixture against RSV was tested in the field in Fujian (Zhangzhou, Longyan), Jiangsu (Guanyun, Yangzhou), Jiangxi and Hunan Provinces, including six host varieties (Table 1). For the control treatment (only sprayed with water), disease indexes were all very high from 2.77 to 11.5. After sprayed with the acetone extract and 8% Ningnanmycin, disease indexes were much lower than those of control treatment. As the dose of the acetone extract increases from 300 to 600 g ha^-1^, disease index gradually decreased and control effect increased. The best antiviral activity was reached at the dose of 600 g ha^-1^, which results in better antiviral effect than 8% Ningnanmycin at 750 g ha^-1^. These results were all similar during all the fields tested, regardless of the host variety used, indicating that the acetone extract is antiviral effective against RSV and widely applicable in different fields and different host varieties.

The acetone extract also show similar and effective antiviral activity against SRBSDV, which was tested in Fujian (Zhangzhou, Longyan), Jiangsu (Guanyun), Jiangxi and Hunan provinces, including 7 host varieties (Table 2). At the dose of 600 g ha^-1^, rice plants in all the fields tested resulted lowest disease index and highest control effect, compared with 8% Ningnanmycin at 750 g ha^-1^, and the acetone extract at 300 and 450 g ha^-1^. These results indicated that the acetone extract also show effective antiviral activity against SRBSDV and is widely applicable in different fields and different host varieties in the south of China.

### Phytotoxic activity and environmental toxicology

The acetone extract was tested for their phytotoxic activity, and showed no phytotoxic activity either in tobacco in the greenhouse or in rice in the field trial.

In Japanese quail, 7-d LD_50_ value of this acetone extract was greater than 66.0 a.i.mg kg^-1^. In *Daphnia magna* 48-h EC_50_ value was 43.1 a.i.mg kg^-1^. In *Scenedesmus obliquus* 72-h EC_50_ value was 9.07 a.i.mg kg^-1^. EC_50_ (96 h) for *Bombyx mori* was 245 a.i.mg kg^-1^. EC_50_ (96 h) for fish was 2.18 a.i.mg kg^-1^. EC_50_ (48 h) for bees was 119 a.i.mg kg^-1^. According to "environmental safety evaluation test guidelines of chemical pesticides" of China, this acetone extract was slightly toxic to Japanese quail, *Daphnia magna*, *Scenedesmus obliquus* and *Bombyx mori*, and moderately toxic to fish and bees. In conclusion, the environmental toxicology testing revealed that this acetone extract is safe enough to be applied in the field and is environmentally friendly.

## Discussion

Gossypol and β-sitosterol were separated from acetone mixture extracted from cottonseed oil sludge and tested for its antiviral effect against TMV in greenhouse, and RSV and SRBSDV in the field trials. As a result, its antiviral effect is better than the commercial agent Ningnanmycin in all three types of viruses including TMV, RSV and SRBSDV, and all infected plant species, indicating that this acetone extract is effectively against a broad spectrum of plant viruses in various plant species including both dicots (tobacco) and monocots (rice). Gossypol and β-sitosterol were seperated from the acetone extract and tested active against TMV. Environmental toxicology showed that this new acetone extract is environmentally friendly and no phytotoxic activity was found in the treated plants.

Gossypol (GOS), a polyphenolic compound isolated from cotton seeds, has been applied as a male contraceptive drug for many years, and also has several new clinical applications including antiviral, antimalarial, and antitumor effects [12–14]. Gossypol, and its derivatives and analogs have been reported as effective agents against human immunodeficiency virus tipe 1 (HIV-1) [15–17], which is proved to be an HIV-1 reverse transcriptase inhibitor by targeting the non-nucleoside inhibitor binding pocket of reverse transcroptase [15]. An et al. [16] also found that the alanine-(-) gossypol derivative, an effective HIV-1 entry inhibitor, can bind to the gp41 hydrophobic pocket and block the formation of the cell fusion-activated gp41 core. In this study, gossypol was found to exhibit effective anti-TMV activity with 54.4% curative effect at 500 μg ml^-1^.

β-sitosterol, the dominant phytosterol with chemical structures similar to that of cholesterol, has been reported to have several medical uses such as hypocholesterolemic activity [18, 19], anti-inflammatory activity [20], induction of apoptosis [21, 22], immunomodulatory activity [23] and anti-oxidant effect [24, 25]. In addition, β-sitosterol isolated from aerial parts of *Cissus sicyoides* showed antibacterial activity against *Bacillus subtilis* with minimal inhibitory concentration (MIC) of 50 μg mL^-1^ [26]. Peres et al. [27] analyzed chemical compositions and antimicrobial activity of *Croton urucurana*, and found. Hex/DCM fraction containing β-sitosterol exhibited the highest inhibitory effect against *Staphylococcus aureus* (0.8 mg mL^-1^). Gebre-Mariam et al. [28] revealed that extracts containing β-sitosterol from *Euclea schimperi* showed antiviral activity against coxsackievirus B3 (CVB3), influenza A virus and herpes simplex virus type 1 Kupka (HSV-1). Alphaamyrin, uvaol, ursolic acid and its lactone, and β-sitosterol were detected in the leaves of *Euclea schimperi* [29]. But there are no relevant reports on antiviral activity of ouabain, amyrin, uvaol, and β-sitosterol. Lin et al. [30] found that β-sitosterol, one of the five major compounds of the *Isatis indigotica* root, inhibited cleavage activities inhibited cleavage activities of the SARS coronavirus 3C-like protease in cell-free and cell-based assays. In this study, we confirmed that β-sitosterol has antiviral properties in a certain degree, although not better than that of gossypol, with 45.6% curative effect at 500 μg mL^-1^ against TMV.

When compared with its individual components (gossypol and β-sitosterol), the extract mixture resulted in the best curative effect (71.2%). In the form of complex mixture, the bioactive compounds may simultaneously function as antiviral activity, dependently or independently with each other, thus reinforcing the inhibiton effect against viruses. Another advantage of mixture of antiviral agents has been discussed by previous researches. One important challenge for any antiviral drugs is the development of resistance by various viruses. In this case, mixture of antiviral compounds is more efficient and practical than the single ones. Because a virus that has developed resistance to a particular drug may not be resistant to other antiviral compounds, which have the potential to possess similar, if not identical, antiviral activities [31].

Natural-product-based antiviral agents have the ability to decompose rapidly, thereby reducing their risk to the environment [32, 33]. In this study, no toxicity was found in tobacco or rice plants. And this acetone extract was slightly toxic to Japanese quail, *Daphnia magna*, *Scenedesmus obliquus* and *Bombyx mori*, and moderately toxic to fish and bees. This environment friendly acetone extract holds potential promise for commecial application in the future. Firstly, from economic considerations, the starting material cottonseed is widely available in China and the cost may be very low. Secondly, cottoseed oil sludge rather than cottonseed oil was served as the raw material, which is the precipitation and enrichment of antiviral effective compounds, so that maximum efforts have been made to improve the concentration of active compounds. Most importantly, the in vivo and field trials demonstrated that this acetone extract was broad-spectrum antiviral, not only against different plant virus (TMV, RSV, SRBSDV), but also more effective in different host varieties from different geographic distributions in China, compared with the commecial agent Ningnanmycin.

In summary, in this study, we firstly discovered acetone extract from cottonseed oil sludge as novel antiviral agent against plant viruses such as TMV, RSV and SRBSDV. Also to our knowledge this is the first report on the application of gossypol and β-sitosterol to plant virus control. Further study will be focused on the antiviral mechanism of these compounds against plant viruses. Hopefully, our results will provide some useful insights for the future virus control strategies.
